# The Cosmetics of the Market

**Published:** 1885-04

**Authors:** 


### THE COSMETICS OF THE MARKET.

In a paper by Dr. James P. Tuttle (*Medical Record*) the popular opinion that cosmetics, if free from lead, are not injurious, is demonstrated to be fallacious.

An analysis of the most largely sold of these compounds reveals the following:

| *Named Preparations.* | *Main Constituents.* |
| --- | --- |
| POWDERS. | |
| Pearl White | Subnitrate bismuth. |
| Flake White | Carbonate lead. |
| Saunders' Face Powder | Oxide of zinc. |
| Complexion Powder | Bismuth subcarbonate. |
| Riker's Face Powder | Calcium and zinc carbonate. |
| LOTIONS. | |
| Circassian Cream | Corrosive sublimate. |
| Kalydor | Corrosive sublimate and potash. |
| Milk of Roses | Corrosive sublimate, rose water and oil of almond. |
| ENAMELS. | |
| Laird's Bloom of Youth | Oxide zinc and calcium. |
| French's Grease Paint | Oxide zinc and calcium. |
| Gouraud's Oriental Cream | *Calomel and water.* |
| Hagan's Magnolia Balm | Oxide zinc, |
| Bradford's Enameline | Oxide zinc. |
| Eugenie's Favorite | Carbonate lead. |
| Snow White Enamel | Carbonate lead. |
| Snow White Oriental Cream | Carbonate lead. |

It will thus be seen that mercury enters largely into the composition of the lotions and enamels as well as zinc. The similar behavior of lead, mercury, zinc and bismuth with the alkali metals found in the blood would in the beginning suggest an analogy in their physiological effects. The acute toxic effects of these drugs are no less similar, and zinc, and probably bismuth, may produce the same general effects.—*New Idea.*

